# Editorial: Digital transformations and the changing nature of work

**DOI:** 10.3389/fsoc.2025.1741944

**Published:** 2025-11-25

**Authors:** Sofia Alexandra Cruz, José Soeiro

**Affiliations:** 1 Faculty of Economics, University of Porto, Porto, Portugal; 2 Department of Sociology, Faculty of Arts and Humanities, University of Porto, Porto, Portugal

**Keywords:** work, digitalization, digital labor platforms, remote work, platform capitalism, labor regulation

Digital transformation of work, the rise of online technologies and virtual employment relations have become a central theme of scholarly and political concern over the past decade (Chen et al., 2024; Crouch, 2018; Drahokoupil, 2021; ILO, 2018, 2024; Ness, 2023; Srnicek, 2016; Vallas and Schor, 2020; Wood et al., 2025). Online labor platforms, algorithmic management, remote work, automation and artificial intelligence are reconfiguring how work is performed, organized, and experienced, giving origin to new inequalities between social groups, genders, regions and countries, but also new business models, subjective agencies and resistances. This shift has intensified in the wake of the COVID-19 pandemic, which has functioned as an accelerant for already ongoing trends that are changing how work is done and what it means to work.

In this context, the Research Topic on “*Digital Transformations and the Changing Nature of Work*” sought to gather diverse contributions that would interrogate these transformations from multiple angles and offer empirical and methodological insights, theoretical frameworks, and grounded case studies from different regions of the world, from the US to the Gulf countries, from southern Europe to Turkey or Brazil. The seven articles included in the current Research Topic offer a nuanced understanding of different aspects of the digital transformations of work, its impacts and challenges.

Dunn et al. provide a longitudinal analysis of the U.S.-based online freelancers, focusing on their experiences both pre- and in-pandemic, and challenge the idea that digital labor markets democratize work or flatten gender inequalities. Instead, the pandemic revealed a regression in gender equity, as women disproportionately absorbed domestic responsibilities and caregiving, leading to reduced work hours and income insecurity. The authors suggest that female online freelancers (and female workers in general) are facing a “double disruption” to their working lives and two generations of progress stands to be lost. Their insights resonate with broader feminist critiques of remote labor and emphasize the need for a gender-sensitive lens in future research and policy options.

Bousrih et al. adopt a macroeconomic perspective to evaluate the effects of digitalization on employment in the Gulf Cooperation Council Countries (GCC), comparing them to some selected advanced countries using an autoregressive distributed mode. Through econometric modeling, they reveal that digitalization has a negative impact on employment in GCC countries, largely due to skill shortages and weak innovation ecosystems. The authors' comparative approach underscores the global unevenness of digital transformations and the critical role of national institutional capacities in mediating their effects, and present recommendations to focus on limiting the digital gap on three main areas: digital skills, research and innovation and the localization of digital products.

Cruz and Gameiro argue for a reconceptualization of digital labor platforms as relational socio-economic systems that involve a triangular relation between platforms, workers, and clients. The authors stress the importance of a service relation approach that embeds the phenomenon in broader consumption practices and managerial logics and in a web of interdependencies between distinctive types of platforms, workers and clients, with considerable differences. Their relational approach opens up new methodological and theoretical pathways for analyzing platform work beyond the binary of control vs. autonomy, shedding light on how workers and clients also shape algorithms in diverse ways.

Sreya et al.'s contribution focuses on the Indian IT sector to explore how remote work and organizational environments affect women's work-life balance. Employing both exploratory and confirmatory factor assessments, the authors identify key factors of satisfaction in women's work-life balance and highlight how psychometric tools can be both reflective of and instrumental. The study explores the differences between beliefs in terms of focus and origin and compares the level of performance of female employees with the acceptance of telework, revealing how support for work-life balance is a critical component of job satisfaction and performance. This work, with reference to the pandemic experience, reinforces the need to examine how digital work environments intersect with gender norms and the lived realities of workers.

Ersöz and Başaran analyze resistance among delivery and transport platform workers in Istanbul. They tackle the diversified labor/capital struggle processes on platforms operating in Istanbul and in the delivery and transportation sector, revealing diverse forms of labor resistance and the struggle around employment status. Their comparative focus emphasizes how resistance is shaped by class dynamics, regulatory frameworks, and local political economies. While delivery workers engage in classic labor struggles for recognition and regulation, taxi drivers' resistance centers on the defense of rent and status. The authors provide an account of how platformization varies by sector, demonstrating that platform labor cannot be understood in isolation from broader socio-political contexts, institutions and actors.

Cruz and Sales Oliveira develop the service relation approach, considering the relation between platforms, workers and clients, and offer a case-study analysis of a Portuguese platform company, Xico's, with a specific business model marked by strong territorial roots, relational proximity and a distinct organizational ethos. The case illustrates how local context, proximity, and a culture of experimentation of the young age founding partners were able to combine the modern logic of platformization with attention to the worker, clients and the territory, in an alternative to standardized international capital-oriented platforms. The article makes it clear that the changing nature of work is not monolithic and can be shaped by alternative logics of workers and community engagement.

Finally, Soeiro et al. develop a comparative approach of labor regulation in Brazil, Portugal and Spain, providing a rich comparative account of regulatory responses to platform labor, including installation process, political debate, positions of different collective actors and also alternative solutions put forward by movements and public policies. They document each country's reality and highlight how different national contexts regionally shape the global phenomenon of platform capitalism. Despite these differences, the study identifies shared patterns of worker resistance, regulatory evasion by platforms, and the emergence of alternative organizational models. The comparative lens proposed is relevant for crafting more just, effective and context specific regulations and alternatives.

In short, the articles in the Research Topic on “*Digital Transformations and the Changing Nature of Work*” paint a complex picture of digital transformations in work and reveal the considerable scholarly interest in the phenomenon of remote work and platforms as two of the most relevant changes both in the nature of work and the organization and division of work. Taken together, the different contributions remind us that digitalization is not a neutral or uniform process, but that it is shaped by gender, age, educational level, class, geography, and different legal and institutional contexts. Digital transformations bring opportunities, challenges, and risks that demand critical study and new regulations, if we want that the future is not only technological but also decent for workers and citizens in general.
